# Shifts of Faecal Microbiota During Sporadic Colorectal Carcinogenesis

**DOI:** 10.1038/s41598-018-28671-9

**Published:** 2018-07-09

**Authors:** Giorgia Mori, Simone Rampelli, Beatrice Silvia Orena, Claudia Rengucci, Giulia De Maio, Giulia Barbieri, Alessandro Passardi, Andrea Casadei Gardini, Giovanni Luca Frassineti, Stefano Gaiarsa, Alessandra M. Albertini, Guglielmina Nadia Ranzani, Daniele Calistri, Maria Rosalia Pasca

**Affiliations:** 1 Department of Biology and Biotechnology “Lazzaro Spallanzani”, University of Pavia, Pavia, Italy; 2 Unit of Microbial Ecology of Health, Department of Pharmacy and Biotechnology, University of Bologna, Bologna, Italy; 3 Biosciences Laboratory, Istituto Scientifico Romagnolo per lo Studio e la Cura dei Tumori (I.R.S.T.), Meldola (FC), Italy; 4 Department of Medical Oncology, Istituto Scientifico Romagnolo per lo Studio e la Cura dei Tumori (IRST) IRCCS, Meldola (FC), Italy; 5 S.C. Microbiologia e Virologia, Fondazione IRCCS Policlinico San Matteo, Pavia, Italy; 6 Dipartimento di Bioscienze, Università degli Studi di Milano, Milan, Italy

## Abstract

Gut microbiota has been implicated in the etiopathogenesis of colorectal cancer. The development of colorectal cancer is a multistep process by which healthy epithelium slowly develops into preneoplastic lesions, which in turn progress into malignant carcinomas over time. In particular, sporadic colorectal cancers can arise from adenomas (about 85% of cases) or serrated polyps through the “adenoma-carcinoma” or the “serrated polyp-carcinoma” sequences, respectively. In this study, we performed 16 S rRNA gene sequencing of bacterial DNA extracted from faecal samples to compare the microbiota of healthy subjects and patients with different preneoplastic and neoplastic lesions. We identified putative microbial biomarkers associated with stage-specific progression of colorectal cancer. In particular, bacteria belonging to the Firmicutes and Actinobacteria phyla, as well as members of the Lachnospiraceae family, proved to be specific of the faecal microbiota of patients with preneoplastic lesions, including adenomas and hyperplastic polyps. On the other hand, two families of the Proteobacteria phylum, Alcaligeneaceae and Enterobacteriaceae, with *Sutterella* and *Escherichia/Shigella* being the most representative genera, appeared to be associated with malignancy. These findings, once confirmed on larger cohorts of patients, can represent an important step towards the development of more effective diagnostic strategies.

## Introduction

Colorectal cancer (CRC) is one of the leading causes of cancer related mortality globally, accounting for about 1.2 million new cases and 690.000 deaths per year^1,2^. CRC is the third most common cancer in men and the second in women; its incidence is rising rapidly in many low-income and middle-income countries, while it is stable in more economically developed countries^1,2^.

CRC arises from a multistep process by which healthy gut epithelium slowly develops into preneoplastic lesions, which in turn progress into malignant carcinomas^3,4^. Most sporadic CRCs follow the conventional “adenoma-carcinoma” sequence, associated with specific mutations occurring at specific progression stages^2,3^. In particular, about 85% of CRCs develop from a benign adenomatous polyp where an *APC* gene mutation is the first pathogenic event^5–10^. Specific adenomas’ features, such as size and histology, allow adenomas’ classification as low- or high-risk adenomas, based on the patients’ likelihood of developing advanced neoplasia during surveillance^3,11^. Recently, molecular advances have identified an alternative pathway of colorectal carcinogenesis involving *BRAF* gene mutations and CpG islands’ hypermethylation. This pathway characterizes the “serrated polyp-carcinoma” sequence in which serrated polyps are the precursor lesion of CRC. Serrated polyps form a heterogeneous group of lesions that includes hyperplastic polyps, a tiny percentage of which progress to malignant cancer^12–14^.

The formation of intestinal preneoplastic lesions and carcinomas are associated with several risk factors, where microbiota dysbiosis (the alteration of the normal microbial community) is one of the most relevant^15–17^.

The microbiota is a complex community of microorganisms that coexist in close association with the host^18^. At present, there is no consensus on the composition of the gut microbiota in preneoplastic lesions or CRC. However, some bacterial species such as *Streptococcus bovis*, *Bacteroides fragilis*, *Enterococcus faecalis*, *Fusobacterium* spp., and *Escherichia coli*, have been correlated with CRC carcinogenesis^16,19^. Gut microbiota could induce CRC carcinogenesis through several mechanisms, including: genotoxin production, modulation of host defences and inflammation pathways, oxidative stress induction and anti-oxidative defence regulation. These mechanisms can lead to genomic instability and epithelial cell proliferation, both underlying colorectal carcinogenic process^11^.

In order to explain how gut microbiota could affect the development of CRC, the “driver-passenger” theory was proposed^16^. This model states that some gut bacteria, called “drivers”, induce epithelial DNA damage and cause inflammation by increasing cell proliferation and/or by producing genotoxic substances that contribute to the accumulation of mutations during tumorigenesis. These changes facilitate the gradual replacement of driver bacteria by “passengers”, with a growth advantage in the tumour microenvironment^16^. CRC progression may be either suppressed by probiotic species or promoted by pathogenic microorganisms. Because of their temporal association with the gut mucosa, driver and passenger bacteria have different roles in CRC progression, but each of them causes alterations during carcinogenesis. Most of the “driver” bacteria may be underrepresented in the adenoma-carcinoma sequence, nonetheless their presence is enough to induce cancerogenesis^16,20^.

The characterization of the microorganisms participating in the genesis and progression of CRC could lead to the prediction of the evolution of pre-cancerous lesions by monitoring the absence of protective bacteria or the presence of possible drivers. Nakatsu and colleagues^21^ tried to identify microbes with potential causative roles in adenoma-carcinoma sequence performing 16 S rRNA gene sequencing on normal mucosae, adenomatous and malignant lesions. In particular, they found a specific enrichment of *Fusobacterium* in CRC lesions and a progressive increasing abundance of *B*. *fragilis* and *Granulicatella* along the adenoma-carcinoma sequence^21^.

Aim of this study was to search for specific microbial patterns that could potentially be used as biomarkers of sporadic CRC carcinogenesis. Accordingly, we analysed and compared the faecal microbiota from healthy controls and from patients with either hyperplastic polyps, low-risk adenomas, high-risk adenomas or adenocarcinomas. Moreover, patients with adenocarcinomas who had received chemotherapy and/or radiotherapy were also investigated in order to assess the influence of treatments on gut microbiota composition.

## Results

### Clinical features of patients

All subjects enrolled in this study had undergone colonoscopy in order to assess the possible presence of colorectal lesions (Table S1). Subjects with negative colonoscopy were recruited as controls (healthy group). The study population (92 individuals, average age = 61.59) was composed of 6 groups: healthy (H = 18; average age = 58.16), hyperplastic polyps (HP = 14; average age = 59.42), low-risk adenomas (LRA = 18; average age = 56.88), high-risk adenomas (HRA = 21; average age = 60.76), adenocarcinomas (ADK = 8; average age = 67.37) and a last group composed of patients with ADK who received chemotherapy and/or radiotherapy treatment (ADK-T = 13; average age = 67) (Table S1).

All clinical data are reported in Table S1, including localisation of colonic lesions and possible additional comorbidities.

### Richness analysis

Illumina 16S rRNA sequencing of the DNA extracted from the 92 faecal samples produced a total of 11.087.753 filtered reads with an average of 120.519 sequences per sample (range: 13.616–442.049). In total, 79.705 operational taxonomic units (OTUs) were delineated at a 97% similarity level.

**Figure 1:**
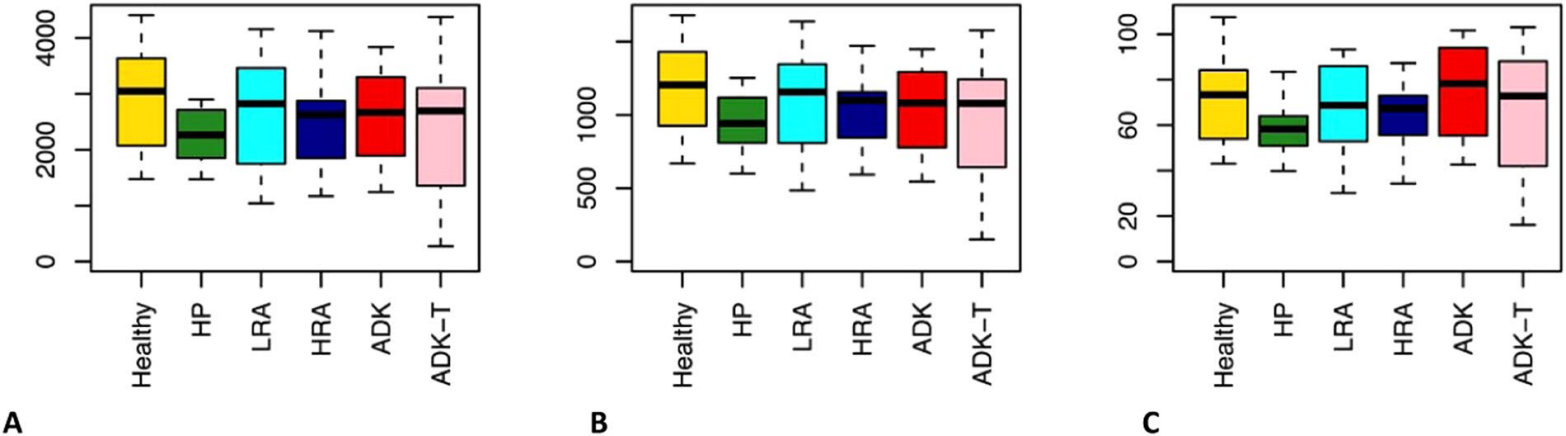
Evaluation of α-diversity in the 5 groups of patients and controls. (**A**) Chao1 measure of microbial richness; (**B**) Observed OTUs; (**C**) Phylogenetic diversity.

Several different metrics were used to calculate α-diversity, including the Chao1 index for microbial richness, OTU species count, and phylogenetic diversity. All measures indicated a comparable microbial richness within the six groups (Fig. 1, Kruskal-Wallis test, *P* > 0.05).

### Main bacterial taxa associated with preneoplastic/neoplastic lesions

Among the identified bacterial OTUs, here we present the most interesting taxa. The *P*-values assigned to each taxa (phylum/family/genus) refer to its significant contribution in every stage of the CRC progression (Figs S2, S3, S4).

Nineteen phyla were detected in patients with preneoplastic/neoplastic lesions and 18 phyla were detected in healthy subjects. In all groups, the five most abundant phyla were the following: Firmicutes, Bacteroidetes, Actinobacteria, Proteobacteria, and Verrucomicrobia (Figs S1; 2).

**Figure 2:**
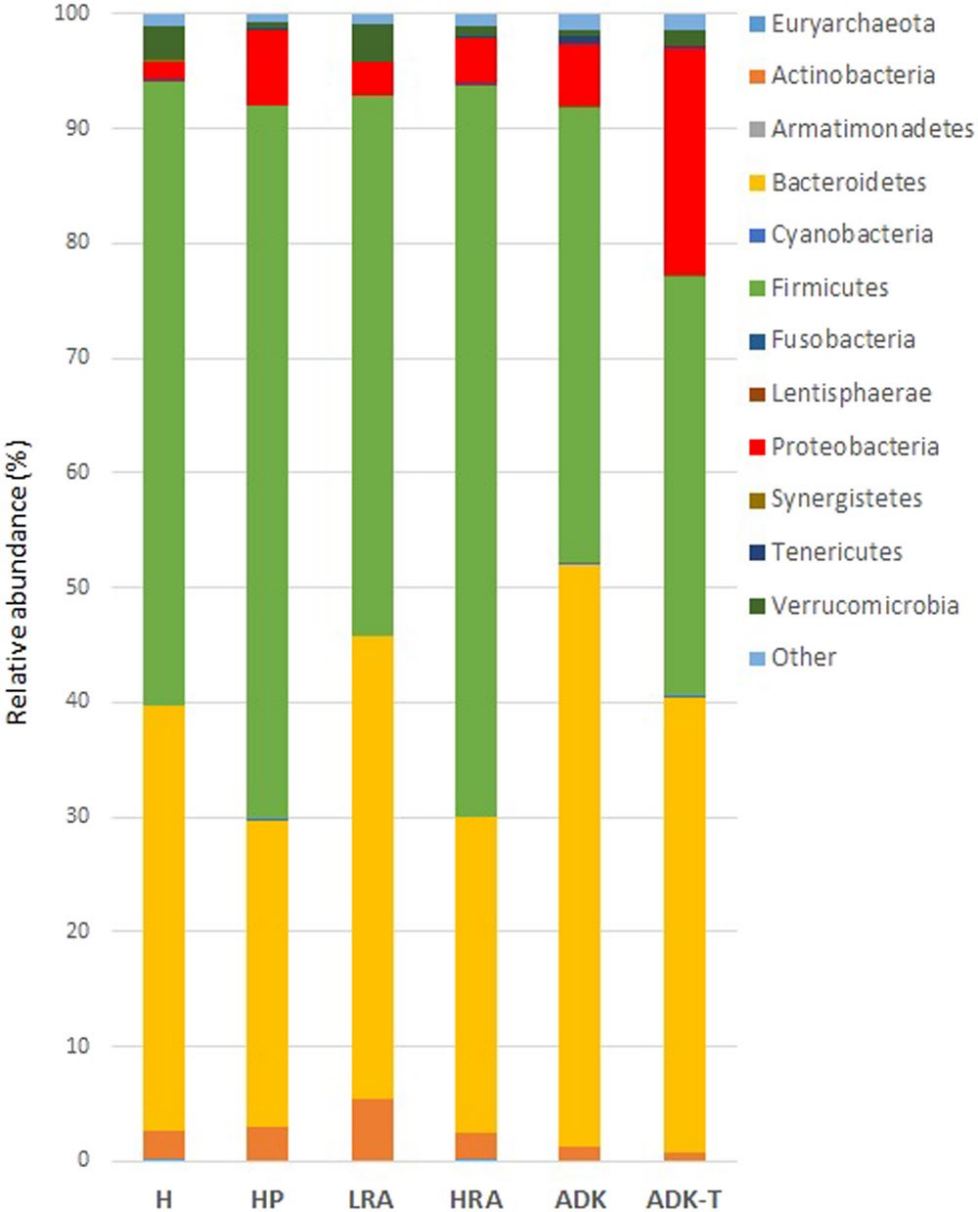
Relative abundance of the main bacterial phyla in faecal microbiota of 7 groups of subjects. Histograms based on the proportion of OTUs per average of each group.

The bacterial flora analysis showed that Firmicutes was the most predominant phylum, contributing with the lowest values in ADK samples (39.65% and 36.49% in ADK and ADK-T, respectively). In healthy subjects and in patients with adenomas and hyperplastic polyps, the abundance of Firmicutes was higher, ranging from 47.14% (LRA) to 62.19% (HP) and 63.71% (HRA) (Figs 2; S2; *P* = 0.009).

The second most dominant phylum was Bacteroidetes with the highest contribution in ADK samples (50.73% and 39.60% in ADK and ADK-T, respectively). A lower contribution was found in healthy subjects (37.13%). In HP, LRA and HRA groups, Bacteroidetes was present at 26.8%, 40.33% and 27.56%, respectively. Indeed, these differences were not significant (Figs 2; S2; *P* = 0.13).

Proteobacteria was the third most abundant phylum with significant richness in ADK samples (5.4% and 19.71% in ADK and ADK-T, respectively). The lowest contribution of this phylum was shown by healthy subjects (1.6%). In the other groups, Proteobacteria phylum was found with the following abundances: 6.57% in HP, 2.83% in LRA and 4.1% in HRA (Figs 2; S2; *P* value = 0.005).

On the contrary, Actinobacteria showed their lowest abundance in ADK patients (1.26% and 0.66% in ADK and ADK-T, respectively) and their highest abundance in H (2.31%), LRA (5.31%) and HP (2.8%) samples. This observation is supported by a *P* value of 0.06 (Figs 2; S2), suggesting a slight trend of increase towards benign lesions and healthy conditions.

Although results were not statistically significant, Verrucomicrobia showed the lowest abundance in ADK samples (0.47% and 1.45% in ADK and ADK-T, respectively) and the highest one in LRA patients (3.3%) (Figs 2; S2; *P* value = 0.9).

Among the poorly represented phyla, Fusobacteria showed low levels in LRA (0.06%) and healthy subjects (0.04%); they were almost absent in HP patients (0.007%) and poorly present in ADK-T group (0.03%), as well as in HRA and ADK samples (0.14% and 0.11%, respectively). In the same way, some authors^22,23^ observed no significant differences in the abundance of *Fusobacterium*, while other authors reported a positive correlation with CRC^21,24–26^.

More than 140 families and 460 genera were identified in the 92 samples.

Among the identified families, the abundance of Lachnospiraceae proved to be quite low in ADK and ADK-T samples (14.2% and 14.6%, respectively) (Fig. S3; *P* value = 0.017). This finding was confirmed by the results obtained at the genus level; the genera *Blautia* (*P* = 0.01) and *Eubacterium hallii* group (*P* = 0.01), both belonging to the Lachnospiraceae family, were also poorly represented in ADK and ADK-T samples (Fig. S4). The same behaviour was followed by other genera, belonging to the Lachnospiraceae family. The relative abundances of these genera, including *Roseburia*, *Dorea*, *Pseudobutyrivibrio*, *Anaerostipes*, *Coprococcus*, and *Fusicatenibacter*, were characterized by different levels of significativity (*P* values in Fig. S4). On the contrary, Lachnospiraceae family was mainly found in HRA and HP samples (31.7% and 29.8%, respectively) (Fig. S4; *P* value = 0.017).

Similarly to Lachnospiraceae, but with data not supported by a strong statistical evidence, Erysipelotrichaceae family was mostly abundant in HRA and HP patients (2% and 1.8%, respectively) (Fig. S4; *P* value = 0.18).

### Structural comparison of gut microbiota between healthy subjects and patients with preneoplastic/neoplastic lesions

Statistical analyses were performed in order to compare the overall structure of faecal microbiota of all considered groups. Wilcoxon non parametric signed-rank test highlighted an enrichment of Proteobacteria phylum in both ADK (*P* = 0.008) and ADK-T (*P* = 0.00004) samples (Table 1). In ADK samples, this result was confirmed by an increment in Enterobacteriaceae (*P* = 0.008) and Alcaligenaceae (*P* = 0.001) families, as well as in *Escherichia-Shigella* (*P* = 0.007) and *Sutterella* (*P* = 0.001) genera. The abundance of Proteobacteria phylum (*P* = 0.00004) in ADK-T was remarkably higher than in the other taxa, but there was not a corresponding increment at the family or genus level (Table 1).

**Table 1:** Wilcoxon Test: comparison of faecal microbiome from healthy subjects with that of the patients’ groups (HP, HRA, ADK and ADK-T).

**Observed differences in microbiome of ADK-T samples**			
	**Healthy** ± **SEM**	**ADK-T** ± **SEM**	***P*** **value**
Phylum: Actinobacteria	2.3 ± 0.6	0.7 ± 0.2	0.004
Phylum: Proteobacteria	1.6 ± 0.6	19.7 ± 6.6	0.00004
Phylum: Unassigned	0.9 ± 0.1	1.3 ± 1.1	0.003
Genus: *Dorea*	0.8 ± 0.1	0.4 ± 0.1	0.006
Genus: *Fusicatenibacter*	1.0 ± 0.3	0.4 ± 0.3	0.008
Genus: *[Eubacterium] halii group*	1.3 ± 0.2	0.5 ± 0.2	0.0007
**Observed differences in microbiome of ADK samples**			
	**Healthy ± SEM**	**ADK ± SEM**	***P*** **value**
Phylum: Proteobacteria	1.6 ± 0.6	5.4 ± 1.3	0.008
Family: Porphyromonadaceae	1.7 ± 0.3	4.6 ± 1.0	0.004
Family: Prevotellaceae	5.0 ± 3.1	8.1 ± 2.7	0.009
Family: Alcaligenaceae	0.2 ± 0.1	1.5 ± 0.6	0.001
Family: Enterobacteriaceae	0.9 ± 0.4	2.8 ± 0.8	0.008
Genus: *Anaerostipes*	0.8 ± 0.2	0.2 ± 0.1	0.001
Genus: *Sutterella*	0.08 ± 0.04	0.7 ± 0.2	0.001
Genus: *Escherichia-Shigella*	0.8 ± 0.3	2.7 ± 0.8	0.007
**Observed differences in microbiome of HRA samples**			
	**Healthy ± SEM**	**HRA ± SEM**	***P*** **value**
Family: Erysipelotrichaceae	0.6 ± 0.2	2.0 ± 0.5	0.0008
Genus: *Blautia*	6.7 ± 2.9	10.8 ± 2.2	0.007
**Observed differences in microbiome of HP samples**			
	**Healthy ± SEM**	**HP ± SEM**	***P*** **value**
Genus: *Prevotella*	4.2 ± 3.0	0.0007 ± 0.0004	0.001

Each group of patients was compared with the group of healthy subjects. Significant P-values are reported. No statistically significant differences were detected between LRA patients and healthy subjects (data not reported).

The Porphyromonadaceae (*P* = 0.004) and Prevotellaceae (*P* = 0.009) (Table 1) families were also highly abundant in ADK patients.

In ADK and ADK-T patients, we observed a significant low abundance of genera belonging to the Lachnospiraceae family, such as *Anaerostipes*, *Dorea*, *Fusicatenibacter* and *[Eubacterium] halii* group. ADK-T patients were also characterized by a significant low presence of members belonging to the Actinobacteria phylum (Table 1).

By Wilcoxon test, no significant differences were observed comparing the faecal microbiome of LRA to that of H subjects (data not shown).

Interestingly, HRA patient samples were featured by an increment of Erysipelotrichaceae family (*P* = 0.0008) and a significant presence of *Blautia* genus (*P* = 0.007), belonging to Lachnospiraceae family (Table 1).

Finally, in HP patients there was the lowest contribution of *Prevotella* genus compared to healthy subjects (4.2 *versus* 0.0007; *P* = 0.001). In order to better understand this result, the relative abundance of *Prevotella* in each of our patients and controls was considered (Table S2). However, the high presence of *Prevotella* in some samples (relative abundance >10%) did not appear to be related to any clinical feature. The presence of *Prevotella* in gut microbiota is commonly associated with a diet rich in carbohydrates and simple sugars. Moreover, *Prevotella* is a well-known dietary fiber fermenter, being quite abundant in some populations such as Hadza hunter-gatherers^27,28^. Moreover, *Prevotella* genus is involved in the production of short chain fatty acids (SCFAs), mainly acetate, the amount and proportion of which has been reported to be biologically relevant^29,30^. Unfortunately, information about patients’ diet was not available. Consequently, we could only hypothesize that the habitual diet of subjects showing an increase in *Prevotella* genus is responsible for its high presence, particularly in those samples in which *Prevotella* contribution is significantly high, such as: H3, H11 and H14 (53.2%, 11.9% and 10.7%, respectively); LRA16 and LRA18 (23.4% and 19.6%); HRA7 and ADK6 (35.8% and 10.7%) (Table S2). It would be extremely helpful, in addition to the clinical features of patients, to have diet information in order to understand the real contribution of *Prevotella*.

**Figure 3:**
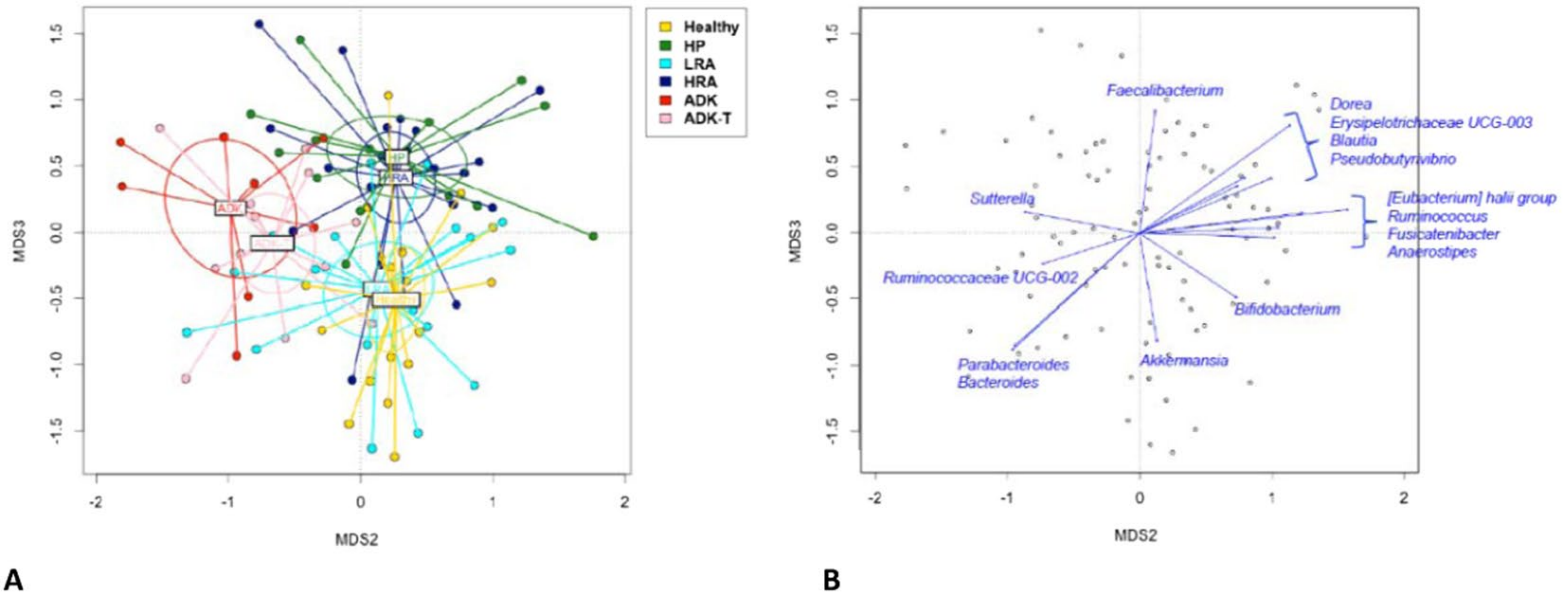
Bacterial taxa differentiate 3 clusters: ADK and ADK patients; HP and HRA patients; LRA patients and H subjects. (**A**) PCoA based on Unweighted UniFrac distances; (**B**) Superimposition of bacterial genera on the PCoA plot.

The principal component analysis (PCoA) based on Unweighted UniFrac distances revealed the formation of 3 different clusters: the first one comprising ADK and ADK-T patients; the second one with HP and HRA individuals; the last one composed of H subjects and LRA patients (Fig. 3A). The superimposition of bacterial genera on the PCoA plot identified the genera involved in these separations. In particular, in ADK and ADK-T patients, the main genera involved were *Sutterella*, Ruminococcaceae UCG-002, *Parabacteroides* and *Bacteroides*. HP and HRA individuals showed a higher contribuition of bacteria, such as *Faecalibacterium*, *Dorea*, *Pseudobutyrivibrio*, *Eubacterium hallii* group, *Ruminococcus*, *Fusicatenibacter* and *Anaerostipes*. The Erysipelotrichaceae family was highly represented in these groups of patients, thus confirming the result obtained with the Wilcoxon test for the HRA group (Table 1). In H and LRA clusters, the discriminant genera were represented by *Bifidobacterium* and *Akkermansia* (Fig. 3).

**Figure 4:**
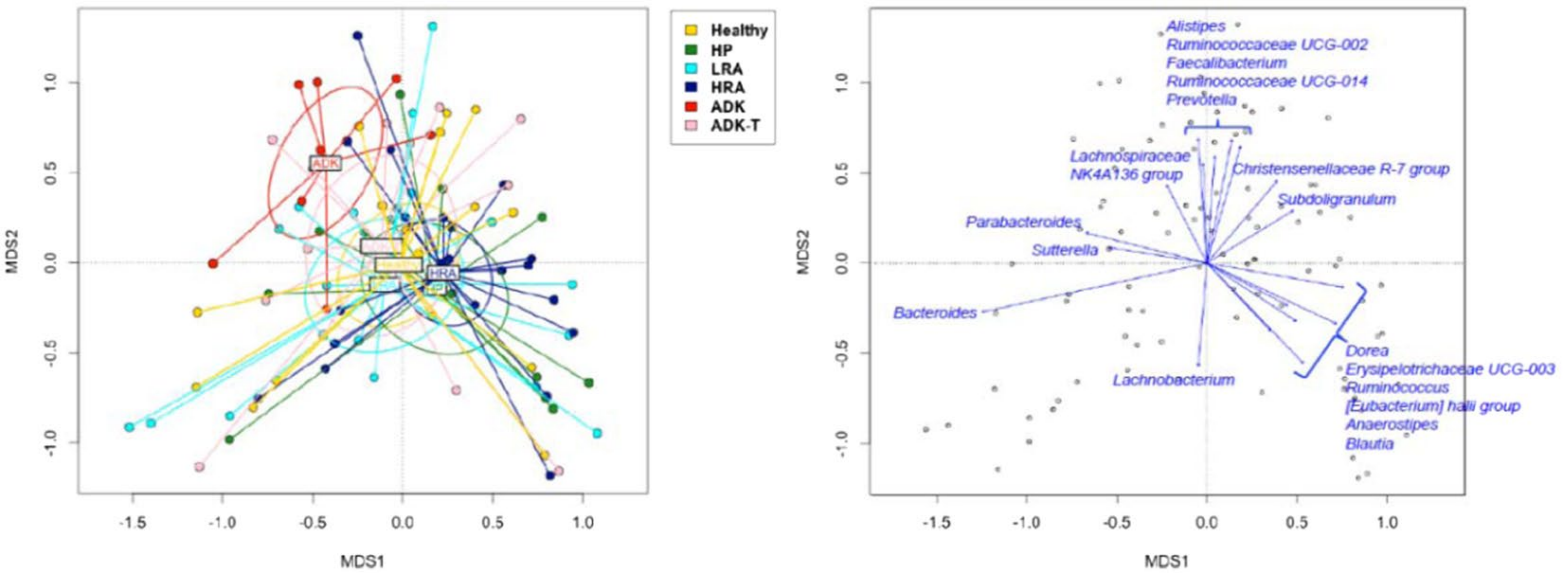
Bacterial taxa abundance differentiates ADK from HP, LRA, HRA and ADK-T patients and healthy subjects. (**A**) PCoA based on Weighted UniFrac distances; (**B**) Superimposition of microbial genera on the PCoA plot.

The PCoA quantitative Weighted UniFrac analysis showed that ADK patients are separated from all the other groups (Fig. 4A), with *Sutterella*, *Parabacteroides* and *Bacteroides* being the main discriminant genera correlated with this separation. Genera *Lachnobacterium*, *Blautia*, *Anaerostipes*, *Dorea* and *Eubacterium hallii* group were prevalent in H, HP, LRA, HRA and ADK-T. In agreement with above reported data, Erysipelotricaceae family was present in this last cluster (Fig. 4B).

### Diversity analysis

**Figure 5:**
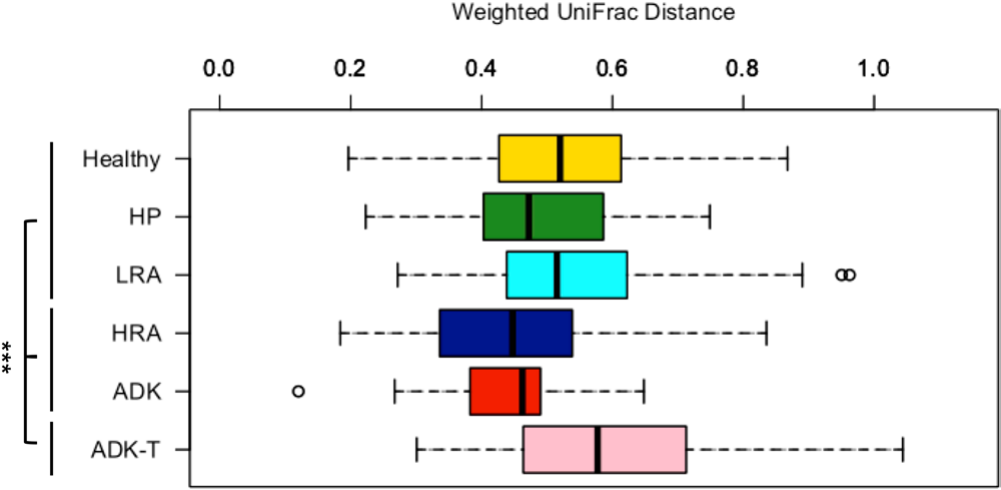
Range of microbial diversity in the investigated groups.

The variation of the Weighted UniFrac MDS3 coordinates between the study groups are reported in Fig. 5. The data were comparable for all the groups except for ADK-T, showing the highest variability (range of microbial diversity: 0.3–1). This result is in agreement with that obtained with the Wilcoxon test in which the significant presence of Proteobacteria was not associated with a significant abundance of specific genera or families (Table 1). Oppositely, the faecal microbiota of ADK-T group was composed of many families and genera belonging to Proteobacteria, such as Alcaligenaceae, Comamonadaceae, Neisseriaceae, Desulfovibrionaceae, Enterobacteriaceae, Moraxellaceae, *Citrobacter*, *Enterobacter*, *Legionella*, *Haemophilus*, *Acinetobacter*, *Pseudomonas*, *Stenotrophomonas*, *Parasutterella*, *Moraxella*, *Aquabacterium*, *Comamonas*, *Delftia*, *Pelomonas*, *Bilophila*, *Desulfovibrio*, *Succinivibrio* and *Cloacibacillus*, all present at very low abundance. Most of these genera are related to nosocomial infections, one of the possible side effects of chemotherapy and/or radiotherapy treatments. Of relevance, ADK-T patients were all characterized by the presence of Proteobacteria, but each of the patients had its specific faecal microbiota profile, probably due to a growth advantage of opportunistic bacteria. Therefore, the absence of a common microbiota response to the treatments suggests that each patient reacts to chemotherapy and/or radiotherapy in an individual way.

Beta diversity is represented and P values are calculated with Wilcoxon Test. Significant results are here reported: (HRA, ADK) *vs* (ADK-T) = 2.568023e-11; (Healthy, HP, LRA) *vs* (ADK-T) = 0.0002791042; (Healthy, HP, LRA) *vs* (HRA, ADK) = 8.430536e-10.

## Discussion

The gut microbiota is composed by a complex community of microorganisms with a critical role in human physiology and metabolism^31–34^. These microorganisms are considered to be one of the main factors influencing the pathogenesis of CRC^35–39^. Differences in the faecal microbiota composition of patients with CRCs or adenomas have been investigated, but available information is insufficient to build a comprehensive picture^40–43^.

In this study, the microbiota composition of LRA, HRA, ADK, ADK-T and HP groups was investigated from faecal samples, using healthy subjects (H) as a control (Table S1).

The average age of our ADK group is higher than those of the preneoplastic groups. This observation is in agreement with the increase in CRC incidence with age^44^: as reported in the AIOM (Italian Association of Medical Oncology) Guidelines^44^, colorectal cancer incidence increases from 8%-4% in men and women younger than 50 years of age, to 14%-17% in subjects older than 70 years of age. Under healthy conditions, the gut microbiota remains relatively stable throughout adulthood. However, the ageing process affects the composition of the microbiota and is associated with loss in microbial diversity and richness^45^. As the average age of our ADK and ADK-T groups is only 5,78–5,41 years higher than the average age of all the subjects analysed in this study, and since the α-diversity analysis revealed a comparable microbial richness within the six groups analysed (Fig. 1), we can hypothesize that the age difference among study groups did not influence our analysis. Moreover, a comparative study addressing differences in gut microbiota along the life-span suggested that age-related changes occur at approximately 75–80 years^46^.

The study of the composition of the faecal bacterial community can be affected by various factors including experimental design and technical procedures (e.g. collection and storage of faecal samples; bacterial DNA extraction; sequencing technology). The methods employed in this work were selected according to protocols that were previously reported to allow a reliable analysis of the faecal microbiota. Samples freezing prior to DNA extraction was earlier shown to result in minimal and non-significant differences in faecal microbiota composition as compared to using freshly collected samples^47,48^. Preservation of the integrity of the faecal microbiota by sample freezing was confirmed by using different sequencing platforms (Roche 454 pyrosequencing, Illumina MiSeq and Ion Torrent)^47–49^ and was shown to be independent of the freezing speed^48^ and of the length of the storage period^47^. In addition, in agreement with recent observations^49^, the use of QIAamp DNA stool kit (Qiagen, Netherlands) allowed extraction of high quality DNA.

**Figure 6:**
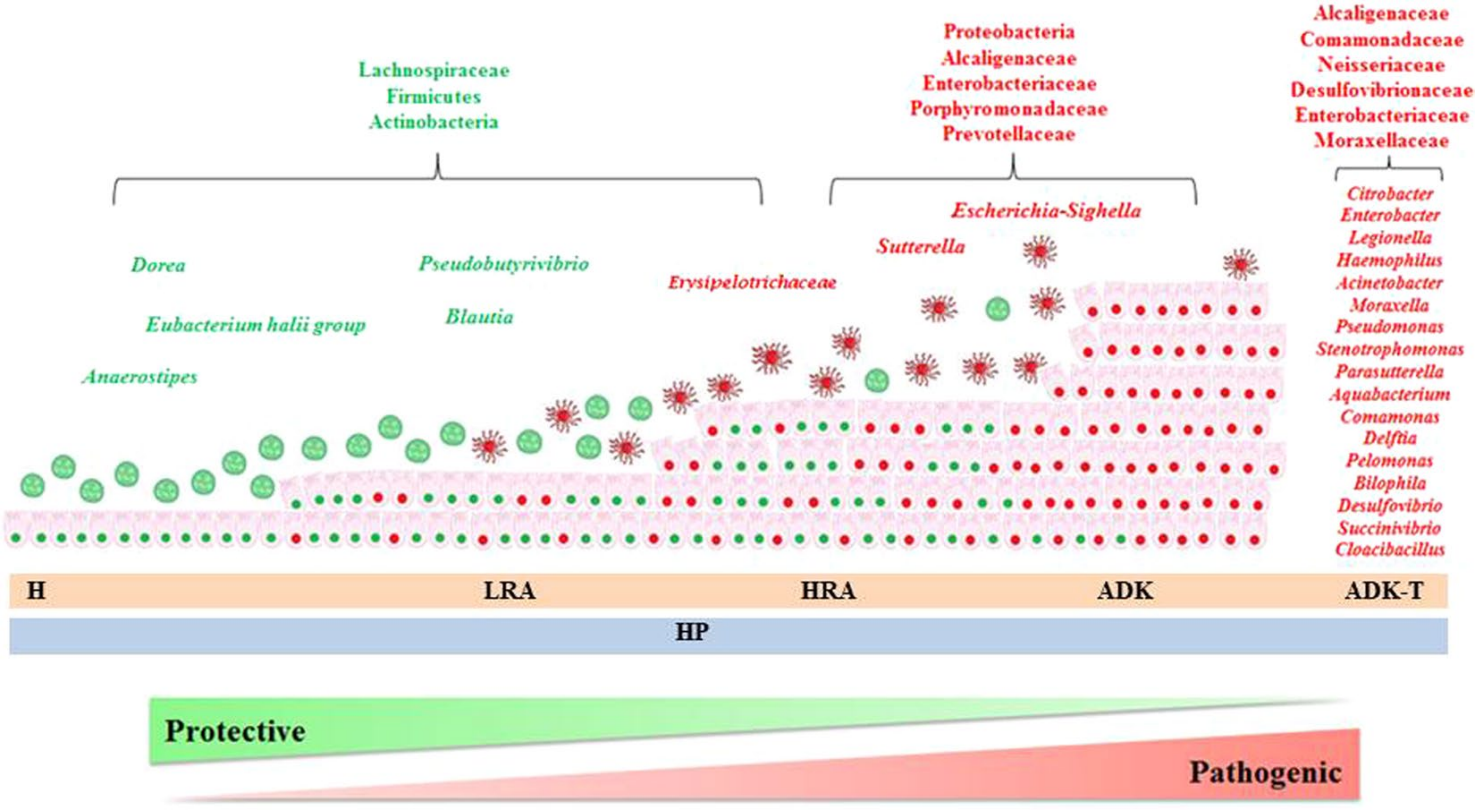
Putative faecal biomarkers of sporadic CRC cancerogenesis.

In this study, we identified microbial associations specific for each group of samples (H, LRA, HRA, ADK, ADK-T and HP); specific microorganisms might represent potential biomarkers of sporadic CRC neoplastic progression (summarised in Fig. 6 and Table 2).

**Table 2:** Putative microbial biomarkers identified in this study.

Phylum	Family	Genus	Purported functions
Actinobacteria			Protective action^20^
Firmicutes	Lachnospiraceae	*Anaerostipes*	Butyrate producer; improve intestinal defense protecting the host^49^
		*Blautia*	Acetate producer; improve intestinal defense protecting the host^27^
		*Dorea*	Protective action^27^
		*Eubacterium hallii* group	Butyrate producer; Protective action^51^
		*Pseudobutyrivibrio*	Butyrate-producer^28^
	Erysipelotrichaceae	—	Potential candidate in the aetiology and progression of colorectal cancer^27^
Bacteroidetes	Porphyromonadaceae	—	Pro-carcinogenic^27,31^
	Prevotellaceae		Pro-carcinogenic^30^
Proteobacteria	Enterobacteriaceae	*Escherichia-Sighella*	Genotoxin-producers; Pro- carcinogenic^27^
	Alcaligenaceae	*Sutterella*	Frequently associated with human diseases^33^

Firmicutes, Bacteroidetes, Actinobacteria and Proteobacteria were the dominant phyla in healthy controls, in agreement with previous studies on gut microflora^18^. A large decrease in Firmicutes and Actinobacteria with concomitant relative expansion of Proteobacteria was observed in patients with ADK. In general, subjects with preneoplastic/neoplastic lesions showed a decrease in the Firmicutes/Bacteroidetes ratio, which might be considered an important marker for intestinal dysbiosis (Figs 2; 6; S2). LRA, HRA and HP samples were characterized by the presence of bacteria belonging to Lachnospiraceae family that, on the contrary, decreased or disappeared in ADK samples (Fig. 6; Table 2).

Schematic representation of CRC progression and associated faecal microorganisms. H = healthy epithelium; LRA = low-risk adenoma; HRA = high-risk adenoma; ADK = adenocarcinoma; ADK-T = adenocarcinoma treated with radio/chemotherapy; HP = hyperplastic polyps. Bacteria specifically identified in each stage of CRC progression are indicated by also taking into account literature data. Green and red indicate protective and pathogenic bacteria, respectively.

As previously described, Actinobacteria and Firmicutes were the most abundant phyla in LRA, HRA and HP groups and decreased in ADK patients (Fig. S2). Similarly, Lachnospiraceae family, belonging to the Firmicutes phylum, was the most abundant in the same groups (Fig. S3). Further evaluating Lachnospiraceae family, *Anaerostipes* significantly decreased in ADK samples (Table 1). This finding is in agreement with previous studies comparing the faecal microbiota of healthy subjects with that of CRC and adenoma patients^19,42^.

Summarizing, Actinobacteria and Firmicutes phyla, as well as bacteria belonging to Lachnospiraceae family, could be considered as good biomarkers specific for H, LRA and HP. It is noteworthy that these three taxa are indicated in literature as bacteria with a putative protective action^20,28,50,51^.

Another possible biomarker of the same groups of patients is represented by Erysipelotrichaceae family, showing its highest contribution in HP and HRA samples (Figs 3; 4; 6), while decreasing in abundance in ADK. The role of Erysipelotrichaceae in inflammation-related disorders of the gastrointestinal tract is known, being these bacteria abundant in the lumen of CRC patients^25,52^. In addition, high levels of Erysipelotrichaceae are related with obesity or, in general, with a high fat diet. Recently, in agreement with our results, Peters and collaborators^42^ found an abundance of Erysipelotrichaceae family in conventional adenomas and in hyperplastic polyps (Fig. 6).

In our study, several genera proved to be overrepresented in ADK patients (Table 1; Figs 3; 4; 6). In particular, the faecal microbiota of ADK patients compared to healthy subjects was enriched in Porphyromonadaceae and Prevotellaceae families, in agreement with previous findings^25^.

Two families belonging to the Proteobacteria phylum, Alcaligeneaceae and Enterobacteriaceae, were dominant in ADK group (Table 1; Figs 3; 4; 6). By considering the Alcaligeneaceae family, *Sutterella* proved to be the most abundant genus (Table 1). Some authors reported that *Sutterella* genus includes commensal bacteria with pro-inflammatory capacities in the gut, proposing its role in the pathogenesis of inflammatory bowel diseases, as well as of some neurological disorders^53^.

Among the Enterobacteriaceae family, the most abundant genus appeared to be *Escherichia/Shigella*. The role of this genus in CRC progression is still under evaluation, being *Escherichia/Shigella* considered both as a driver and as a passenger^16,54^. In our study, *Escherichia/Shigella* was prevalent in the ADK group.

Regarding the ADK group, Alcaligenaceae and Enterobacteriaceae families, with their most representative genera (*Sutterella* and *Escherichia/Shigella*, respectively) could be consider as potential biomarkers of progression to malignancy.

In the ADK-T group, we found the highest diversity in microorganisms belonging to the Proteobacteria phylum (*Citrobacter*, *Enterobacter*, *Legionella*, *Haemophilus*, *Acinetobacter*, *Pseudomonas*, *Stenotrophomonas*, *Parasutterella*, *Moraxella*, *Aquabacterium*, *Comamonas*, *Delftia*, *Pelomonas*, *Bilophila*, *Desulfovibrio*, *Succinivibrio*, *Cloacibacillus*). This is in line with the possible presence of nosocomial infections caused by the treatments (chemotherapy and/or radiotherapy). Of relevance, ADK patients treated with chemotherapy and/or radiotherapy were characterized by the absence of a specific faecal microbiota, suggesting heterogeneous and individual responses to the treatments. Some authors reported that chemotherapy and radiotherapy are associated with reduced diversity in gut microbiota and with changes in its composition^55^. Montassier and collaborators^56^ identified microbes that changed during chemotherapy independently from other additional factors; faecal samples collected after chemotherapy were characterized by an increase of *Enterococcaceae* and *Enterobacteriaceae*, and a decrease of *Ruminococcaceae*, *Lachnospiraceae* and *Bifidobacterium*^57^.

Recently, Sze and collaborators (2018)^57^ tested the composition of faecal microbiota before and after the treatments for adenomas, advanced adenomas and carcinomas. The aim of these authors was to understand how faecal microbiota changes after a long period subsequent the treatment. The authors found that there were no significant differences between pre- and post-treatment samples from patients with adenoma and advanced adenoma; for carcinomas, not only changes were found in pre- *versus* post-treatment samples, but also these changes were toward a more normal microbiota^57^.

In our study, we investigated the gut microbiota composition of ADK-T group patients that were currently under treatment, finding that our ADK-T group was characterized by a change in the faecal microbiota, including an increased microbial diversity, likely due to nosocomial infections (Figs 5; 6). Accordingly, it was impossible to identify a microbial biomarker specific for this group of patients.

To the best of our knowledge, this is the first analysis to evaluate in parallel the faecal microbiota composition of healthy subjects and patients with ADK/ADK-T, LRA/HRA and HP.

In their work, Nakatsu and collaborators^21^ found different microbial associations in mucosal biopsy samples at each stage of adenoma-carcinoma sequence, whilst they did not investigate samples from serrated polyp-carcinoma sequence. As previously shown^58^, the microbiota composition of mucosal and faecal samples from CRC patients differs significantly. For example, in CRC mucosal samples Nakatsu and collaborators found an abundance of *Fusobacterium*, *B*. *fragilis* and *Granulicatella*. In our work, ADK samples were characterized by very low levels of *Fusobacterium* and *Granulicatella* (0.08% and 0.025%, respectively), even if this is the stage in which they are most abundant.

With this study, we compared the faecal microbiota composition of patients with preneoplastic and neoplastic lesions with that of healthy subjects and we identified specific microbial biomarkers associated with each carcinogenic stage. Moreover, the contribution of chemoterapy/radiotherapy was investigated, highlighting the great abundance of opportunistic pathogens.

The identification of bacterial communities that are specific of preneoplastic/neoplastic lesions can be regarded as an important step towards the development of more effective diagnostic strategies. Additional work should be done not only by analysing patients at different risk of CRC progression over a prolonged period, but also a larger cohort of patients, mucosal tissues analysis and a metagenomic approach should be considered. In this way it would be possible to confirm the investigated potential biomarkers of CRC progression and their possible causative association with CRC carcinogenesis.

## Methods

### Study population and stool samples

For this study, we recruited patients diagnosed with hyperplastic polyps (HP), low-risk adenomas (LRA), high-risk adenomas (HRA) and adenocarcinomas (ADK).

All subjects were recruited at the Istituto Scientifico Romagnolo per lo Studio e la Cura dei Tumori (IRST) of Meldola (FC, Italy) between 2013 and 2015. Enrolled patients were a subgroup of the protocol IRSTB002, approved by the Ethic Committee of IRST - IRCCS AVR (25/10/2012, ver.1). All methods were performed in accordance with relevant guidelines and regulations and written informed consent was obtained from all patients. The Biosciences Laboratory of IRST, in collaboration with the Cancer Prevention Services of the Local Health Authority of Romagna (Forlì, Cesena and Ravenna, Italy), is responsible for identifying genetic factors involved in predisposition to cancer and for activating appropriate prevention strategies for individuals at risk. The individuals who are candidates for genetic testing are essentially those with a suspected hereditary predisposition to the development of specific cancers (e.g.: Hereditary Breast and Ovarian Cancer, Hereditary diffuse gastric cancer, Li-Fraumeni Syndrome, Neurofibromatosis Type II and Lynch syndrome). None of the patients included in this study met the criteria for genetic counselling/ genetic testing: early-onset patients and subjects with family history of cancer were excluded from this analysis.

Eligible participants were individuals 40–80 years old who recently underwent colonoscopy. Hyperplastic polyps, adenomas, and adenocarcinomas were identified during colonoscopy and confirmed by the pathologist. Subjects with histologically confirmed normal biopsies were included in the control group (18 subjects).

The results of endoscopic and histological examination were the following: 21 subjects were diagnosed with ADK, 21 with HRA, 18 with LRA, 14 with HP.

Preneoplastic lesions were classified as low- or high-risk according to National Comprehensive Cancer Network guidelines^59^. Briefly, LRA were defined as 1–2 adenomas with size <10 mm; HRA were indicated as 3 or more adenomas with size ≥10 mm.

Stool specimens were collected by each participant about 1–2 days before the bowel preparation for the colonoscopy examination. Faecal samples were immediately stored at 4 °C and brought to the Bioscience Laboratory of IRST (Meldola, Italy) within 24–48 h. The stool samples were suspended in Olson buffer (0.5 mol/L Tris HCl; 0.15 mol/L EDTA, 10 mmol/L NaCl, PH = 9)^60^ and stored at −80 °C. An aliquot of each sample was shipped in dry ice within a time frame of 3 months to the Department of Biology and Biotechnology “Lazzaro Spallanzani” (University of Pavia, Italy) where it is stored at −80 °C until DNA extraction. All samples (included control samples) were processed in the same way.

### Clinical data collection

Demographic information and clinic data are reported in Table S1. Moreover, the localization of colonic lesion for each patient is specified in Table S1. For each participant, data about comorbidities were also recorded. Treatment with chemotherapy/radiotherapy was specifically assessed, given its possible interaction with gut microbiota.

### Extraction of nucleic acids for microbiome analysis

DNA was extracted from faeces using QIAamp DNA stool kit (Qiagen, Netherlands) according to the specifications of the manufacturers. The V4 region of 16 rRNA gene was amplified using the 515 F forward primer (GTGYCAGCMGCCGCGGTAA) and barcoded 806 R reverse primers (GGACTACNVGGGTWTCTAAT) as previously described^61,62^. Sequencing was performed on the Illumina MiSeq platform, and paired-end reads of 250b in length in each direction were generated producing a total of 11.087.753 filtered reads from 92 high-quality samples (6 samples were removed because of technical failures). The reads were pre-processed and analyzed with the Qiime pipeline for taxonomic composition, alpha diversity, and beta diversity analysis^61,63^. Alpha-diversity and rarefaction plots were computed on the normalized OTU table using three different metrics: PD whole tree, chao1 and observed species. Weighted and unweighted UniFrac distances were used to perform PCoA.

### Biostatistics

The R packages Stats and Vegan (http://www.cran.r-project.org/package=vegan) were used to perform statistical analysis. In particular, to compare gut microbiome structure among different groups for α and β diversity, we used a Wilcoxon-signed rank test and a Kruskal-Wallis test. Data separation in the PCoA was tested using a permutation test with pseudo F-ratios (function Adonis in the Vegan package). Significant differences in phylum or family or genus-level abundance between groups, were assessed by Mann–Whitney U-tests, and corrected for multiple comparisons using the Benjamini–Hochberg method when appropriate (Table 1). False discovery rate (FDR) <0.05 was considered as statistically significant.

## Electronic supplementary material

Supplementary material

## References

[1] WHO. World Cancer Report 2014 (2014).

[2] Armaghany T, Wilson JD, Chu Q, Mills G (2012). Genetic alterations in colorectal cancer. Gastrointest Cancer Res..

[3] Calderwood AH, Lasser KE, Roy HK (2016). Colon adenoma features and their impact on risk of future advanced adenomas and colorectal cancer. World J Gastrointest Oncol..

[4] Walther A (2009). Genetic prognostic and predictive markers in colorectal cancer. Nat Rev Cancer..

[5] Cottrell S, Bicknell D, Kaklamanis L, Bodmer WF (1992). Molecular analysis of APC mutations in familial adenomatous polyposis and sporadic colon carcinomas. Lancet..

[6] Hokazono K (2014). A CpG island methylator phenotype of colorectal cancer that is contiguous with conventional adenomas, but not serrated polyps. Oncol. Lett..

[7] Setaffy L, Langner C (2015). Microsatellite instability in colorectal cancer: clinicopathological significance. Pol. J. Pathol..

[8] Fearon ER, Vogelstein B (1990). A genetic model for colorectal tumorigenesis. Cell..

[9] Pino MS, Chung DC (2010). The chromosomal instability pathway in colon cancer. Gastroenterology..

[10] Vogelstein B (1988). Genetic alterations during colorectal-tumor development. N Engl J Med.

[11] Rex DK (2017). Colorectal Cancer Screening: Recommendations for Physicians and Patients From the U.S. Multi-Society Task Force on Colorectal Cancer. Gastroenterology..

[12] Yamane L, Scapulatempo-Neto C, Reis RM, Guimarães DP (2014). Serrated pathway in colorectal carcinogenesis. World J Gastroenterol..

[13] Leggett B, Whitehall V (2010). Role of the serrated pathway in colorectal cancer pathogenesis. Gastroenterology..

[14] Huang CS, O’brien MJ, Yang S, Farraye FA (2004). Hyperplastic polyps, serrated adenomas, and the serrated polyp neoplasia pathway. Am J Gastroenterol..

[15] Sears CL, Garrett WS (2014). Microbes, microbiota, and colon cancer. Cell Host Microbe..

[16] Tjalsma H, Boleij A, Marchesi JR, Dutilh BE (2012). A bacterial driver-passenger model for colorectal cancer: beyond the usual suspects. Nat. Rev. Microbiol..

[17] Wang T (2012). Structural segregation of gut microbiota between colorectal cancer patients and healthy volunteers. ISME J..

[18] Human Microbiome Project Consortium. Structure, function and diversity of the healthy human microbiome. *Nature*. **486**, 207–14, 10.1038/nature11234 (2012). 10.1038/nature11234 PMC3564958 22699609

[19] Gagnière J (2016). Gut microbiota imbalance and colorectal cancer. World J. Gastroenterol..

[20] Yu YN, Fang JY (2015). Gut microbiota and colorectal cancer. Gastrointest Tumors..

[21] Nakatsu G (2015). Gut mucosal microbiome across stages of colorectal carcinogenesis. Nat Commun..

[22] Lu Y (2016). Mucosal adherent bacterial dysbiosis in patients with colorectal adenomas. Sci Rep..

[23] Russo E (2018). Preliminary Comparison of Oral and Intestinal Human Microbiota in Patients with Colorectal Cancer: A Pilot Study. Front Microbiol..

[24] Shang FM, Liu HL (2018). *Fusobacterium nucleatum* and colorectal cancer: A review. World J Gastrointest Oncol..

[25] Baxter NT, Zackular JP, Chen GY, Schloss PD (2014). Structure of the gut microbiome following colonization with human feces determines colonic tumor burden. Microbiome..

[26] Eklöf V (2017). Cancer-associated fecal microbial markers in colorectal cancer detection. Int J Cancer..

[27] Wu GD (2011). Linking long-term dietary patterns with gut microbial enterotypes. Science..

[28] Schnorr SL (2014). Gut microbiome of the Hadza hunter-gatherers. Nat Commun.

[29] Chen T (2017). Fiber-utilizing capacity varies in *Prevotella*- *versus Bacteroides*-dominated gut microbiota. Sci Rep..

[30] Koh A (2016). From Dietary Fiber to Host Physiology: Short-Chain Fatty Acids as Key Bacterial Metabolites. Cell..

[31] Ursell LK, Metcalf JL, Parfrey LW, Knight R (2012). Defining the human microbiome. Nutr. Rev..

[32] Geuking MB, Köller Y, Rupp S, McCoy KD (2014). The interplay between the gut microbiota and the immune system. Gut Microb..

[33] Chung H, Kasper DL (2010). Microbiota-stimulated immune mechanisms to maintain gut homeostasis. Curr Opin Immunol..

[34] Krishnan S, Alden N, Lee K (2015). Pathways and functions of gut microbiota metabolism impacting host physiology. Curr Opin Biotechnol..

[35] Nagao-Kitamoto H, Kitamoto S, Kuffa P, Kamada N (2016). Pathogenic role of the gut microbiota in gastrointestinal diseases. Intest Res..

[36] Tomasello G (2014). Dismicrobism in inflammatory bowel disease and colorectal cancer: Changes in response of colocytes. World J. Gastroenterol..

[37] Wu N (2013). Dysbiosis signature of fecal microbiota in colorectal cancer patients. Microb Ecol..

[38] Sheflin AM, Whitney AK, Weir TL (2014). Cancer-promoting effects of microbial dysbiosis. Curr Oncol Rep..

[39] Wang T (2012). Structural segregation of gut microbiota between colorectal cancer patients and healthy volunteers. ISME J..

[40] Feng Q (2015). Gut microbiome development along the colorectal adenoma-carcinoma sequence. Nat. Commun..

[41] Hale VL (2017). Shifts in the fecal microbiota associated with adenomatous polyps. Cancer Epidemiol. Biomarkers Prev..

[42] Peters BA (2016). The gut microbiota in conventional and serrated precursors of colorectal cancer. Microbiome..

[43] Rosenberg DW (2007). Mutations in BRAF and KRAS differentially distinguish serrated versus non-serrated hyperplastic aberrant crypt foci in humans. Cancer Res..

[44] AIOM (Associazione Italiana di Oncologia Medica). I numeri del cancro in italia 2017. http://www.aiom.it/fondazione-aiom/aiom-airtum-numeri-cancro-2017/1,3021,1 (2017).

[45] García-Peña C, Álvarez-Cisneros T, Quiroz-Baez R, Friedland RP (2017). Microbiota and Aging. A Review and Commentary. Arch Med Res..

[46] Biagi E (2010). Through ageing, and beyond: gut microbiota and inflammatory status in seniors and centenarians. PLoS One..

[47] Carroll IM, Ringel-Kulka T, Siddle JP, Klaenhammer TR, Ringel Y (2012). Characterization of the fecal microbiota using high-throughput sequencing reveals a stable microbial community during storage. PLoS One.

[48] Fouhy F (2015). The effects of freezing on faecal microbiota as determined using MiSeq sequencing and culture-based investigations. PLoS One.

[49] Panek M (2018). Methodology challenges in studying human gut microbiota - effects of collection, storage, DNA extraction and next generation sequencing technologies. Sci Rep.

[50] Sagheddu V, Patrone V, Miragoli F, Puglisi E, Morelli L (2016). Infant early gut colonization by Lachnospiraceae: high frequency of Ruminococcus gnavus. Front Pediatr..

[51] Dinh DM (2015). Intestinal microbiota, microbial translocation, and systemic inflammation in chronic HIV infection. J Infect Dis..

[52] Kaakoush NO (2015). Insights into the Role of Erysipelotrichaceae in the Human Host. Front Cell Infect Microbiol..

[53] Hiippala K, Kainulainen V, Kalliomäki M, Arkkila P, Satokari R (2016). Mucosal Prevalence and Interactions with the Epithelium Indicate Commensalism of *Sutterella* spp. Front Microbiol..

[54] Tomkovich S (2017). Locoregional effects of microbiota in a preclinical model of colon carcinogenesis. Cancer Res..

[55] Roy, S., & Trinchieri, G. Microbiota: a key orchestrator of cancer therapy. *Nat*. *Rev*. *Cancer*. In press; 10.1038/nrc.2017.13 (2017). 10.1038/nrc.2017.13 28303904

[56] Montassier E (2015). Chemotherapy-driven dysbiosis in the intestinal microbiome. Aliment. Pharmacol. Ther..

[57] Sze MA, Baxter NT, Ruffin MT, Rogers MAM, Schloss PD (2017). Normalization of the microbiota in patients after treatment for colonic lesions. Microbiome.

[58] Flemer B (2017). Tumour-associated and non-tumour-associated microbiota in colorectal cancer. Gut..

[59] NCCN Guidelines. https://www.nccn.org/professionals/physician_gls/f_guidelines.asp (2018).

[60] Olson J, Whitney DH, Durkee K, Shuber AP (2005). DNA stabilization is critical for maximizing performance of fecal DNA-based colorectal cancer tests. Diagn Mol Pathol..

[61] Caporaso JG (2012). Ultra-high-throughput microbial community analysis on the Illumina HiSeq and MiSeq platforms. The ISME journal.

[62] Segata N (2016). The reproductive tracts of two malaria vectors are populated by a core microbiome and by gender- and swarm-enriched microbial biomarkers. Sci Rep..

[63] Caporaso JG (2010). QIIME allows analysis of high-throughput community sequencing data. Nature methods.

