# Supplementary material for: Shifts of Faecal Microbiota During Sporadic Colorectal Carcinogenesis
Source: Sci Rep. 2018 Jul 9;8:10329. doi: 10.1038/s41598-018-28671-9 (PMC6037773; doi:10.1038/s41598-018-28671-9)
Supplement: Supplementary file 1 — Supplementary material [file 41598_2018_28671_MOESM1_ESM.doc]

**Supplementary information**

**Title: SHIFTS OF FAECAL MICROBIOTA DURING SPORADIC COLORECTAL CARCINOGENESIS**

**Giorgia Mori**^1,*^**, Simone Rampelli**^2^**, Beatrice Silvia Orena**^1^**, Claudia Rengucci**^3^**, Giulia De Maio**^3^**, Giulia Barbieri**^1^**, Alessandro Passardi**^4^**, Andrea Casadei Gardini**^4^**, Giovanni Luca Frassineti**^4^**, Stefano Gaiarsa**^5,6^**, Alessandra M. Albertini**^1^**, Guglielmina Nadia Ranzani**^1^**, Daniele Calistri**^3^**, Maria Rosalia Pasca**^1*^

^1^Department of Biology and Biotechnology “Lazzaro Spallanzani”, University of Pavia, Pavia, Italy; ^2^Unit of Microbial Ecology of Health, Department of Pharmacy and Biotechnology, University of Bologna, Bologna, Italy; ^3^Biosciences Laboratory, Istituto Scientifico Romagnolo per lo Studio e la Cura dei Tumori (I.R.S.T.), Meldola (FC), Italy; ^4^Department of Medical Oncology, Istituto Scientifico Romagnolo per lo Studio e la Cura dei Tumori (IRST) IRCCS, Meldola (FC), Italy; ^5^S.C. Microbiologia e Virologia, Fondazione IRCCS Policlinico San Matteo, Pavia, Italy; ^6^Dipartimento di Bioscienze, Università degli Studi di Milano, Milan, Italy.

| **Patient ID** | **Sex** | **Age at diagnosis** | **Lesions location** | **Risk classification** | **Comorbidities** | **Chemotherapy** | **Radiotherapy** |
| --- | --- | --- | --- | --- | --- | --- | --- |
| H1 | F | 50 | / | Healthy | Hemorrhoids GIII. | / | / |
| H2 | M | 50 | / | Healthy | Hemorrhoids GI. | / | / |
| H3 | M | 67 | / | Healthy | Hemorrhoids GI. | / | / |
| H4 | M | 59 | / | Healthy | Hemorrhoids GII. | / | / |
| H5 | F | 61 | / | Healthy | Chronic obstructive pulmonary disease (COPD); hypertension. | / | / |
| H6 | M | 57 | / | Healthy | Angiodysplasia. | / | / |
| H7 | F | 61 | / | Healthy | Hemorrhoids GI. | / | / |
| H8 | F | 50 | / | Healthy | Hemorrhoids GI; Treatment for breast cancer prevention. | / | / |
| H9 | M | 69 | / | Healthy | Diverticulosis; Hypertension; metabolic syndrome. | / | / |
| H10 | M | 59 | / | Healthy | Hypertension. | / | / |
| H11 | F | 59 | / | Healthy | Hemorrhoids; colon diverticulosis . | / | / |
| H12 | M | 66 | / | Healthy | Hypertension. | / | / |
| H13 | F | 32 | / | Healthy | Hemorrhoids GII. | / | / |
| H14 | F | 68 | / | Healthy | Hypertension; dyslipidemia. | / | / |
| H15 | F | 56 | / | Healthy | / | / | / |
| H16 | M | 80 | / | Healthy | Hypertension; depression; dyslipidemia. | / | / |
| H17 | M | 58 | / | Healthy | Hypertension. | / | / |
| H18 | F | 45 | / | Healthy | Hypothyroidism. | / | / |
| HP1 | M | 65 | Descending colon | Hyperplastic polyps | Diverticulosis; dyslipidemia (high triglycerides). | / | / |
| HP2 | M | 63 | Ascending colon | Hyperplastic polyps | Hemorrhoids GII; Hypothyroidism. | / | / |
| HP3 | M | 65 | Descending colon | Hyperplastic polyps | Colon diverticulosis; Hemorrhoids GI. | / | / |
| HP4 | M | 60 | Descending colon | Hyperplastic polyps | Hemorrhoids GIII; Hypertension. | / | / |
| HP5 | F | 51 | Descending colon | Hyperplastic polyps | / | / | / |
| HP6 | M | 59 | Descending colon | Hyperplastic polyps | Colon diverticulosis; Hemorrhoids GII. | / | / |
| HP7 | M | 60 | Ascending colon | Hyperplastic polyps | Hypertension; lymphoma. | / | / |
| HP8 | M | 57 | Descending colon | Hyperplastic polyps | Hypertension; initial metabolic syndrome. | / | / |
| HP9 | M | 52 | Descending colon | Hyperplastic polyps | Hemorrhoids GI. | / | / |
| HP10 | M | 53 | Transverse colon | Hyperplastic polyps | / | / | / |
| HP11 | M | 63 | Descending colon | Hyperplastic polyps | Colon diverticulosis. | / | / |
| HP12 | M | 60 | Descending colon | Hyperplastic polyps | Dyslipidemia. | / | / |
| HP13 | M | 59 | Ascending colon | Hyperplastic polyps | Hemorrhoids GI; colitis of ascending colon; Syndrome of Von Willebrand. | / | / |
| HP14 | F | 65 | Ascending colon | Hyperplastic polyps | Hemorrhoids GI; colitis of ascending colon. Hypothyroidism. | / | / |
| LRA1 | F | 64 | Descending colon | Low Risk adenomas | Hemorrhoids GI; dyslipidemia (high triglycerides). | / | / |
| LRA2 | M | 53 | Transverse colon | Low Risk adenomas | Hemorrhoids | / | / |
| LRA3 | M | 50 | Descending colon | Low Risk adenomas | Low-calorie and low-fat diet. | / | / |
| LRA4 | M | 59 | Descending colon | Low Risk adenomas | Hypertension; gout. | / | / |
| LRA5 | M | 57 | Descending colon | Low Risk adenomas | Hemorrhoids GI; colon diverticulosis. | / | / |
| LRA6 | M | 55 | Transverse colon | Low Risk adenomas | Hemorrhoids GIII. | / | / |
| LRA7 | F | 56 | Ascending colon | Low Risk adenomas | Colon diverticulosis; hemorrhoids GI. | / | / |
| LRA8 | M | 58 | Descending colon | Low Risk adenomas | Colon diverticulosis. | / | / |
| LRA9 | M | 53 | Transverse colon | Low Risk adenomas | / | / | / |
| LRA10 | M | 53 | Ascending colon | Low Risk adenomas | Gonalgia. | / | / |
| LRA11 | M | 55 | Descending colon | Low Risk adenomas | / | / | / |
| LRA12 | M | 59 | Right colon | Low Risk adenomas | Dyslipidemia; ischemic heart disease. | / | / |
| LRA13 | M | 57 | Descending colon | Low Risk adenomas | / | / | / |
| LRA14 | M | 53 | Descending colon | Low Risk adenomas | Colon diverticulosis; hemorrhoids GII; hypertension; dyslipidemia. | / | / |
| LRA15 | M | 63 | Descending colon | Low Risk adenomas | / | / | / |
| LRA16 | M | 67 | Ascending colon | Low Risk adenomas | / | / | / |
| LRA17 | F | 51 | Descending colon | Low Risk adenomas | Colon diverticulosis. | / | / |
| LRA18 | M | 61 | Descending colon | Low Risk adenomas | / | / | / |
| HRA1 | M | 57 | Descending colon | High Risk adenomas | / | / | / |
| HRA2 | M | 63 | Descending colon | High Risk adenomas | / | / | / |
| HRA3 | M | 63 | Ascending colon | High Risk adenomas | Hemorrhoids GI; psoriatic oligoarthritis. | / | / |
| HRA4 | F | 64 | Descending colon | High Risk adenomas | / | / | / |
| HRA5 | M | 65 | Descending colon | High Risk adenomas | Hemorrhoids GII. | / | / |
| HRA6 | M | 60 | Ascending colon | High Risk adenomas | Hemorrhoids GII. | / | / |
| HRA7 | M | 61 | Descending colon | High Risk adenomas | Colon diverticulosis; Hemorrhoids GII; Ischemic heart disease. | / | / |
| HRA8 | M | 67 | Descending colon | High Risk adenomas | Hypertension. | / | / |
| HRA9 | M | 54 | Ascending colon | High Risk adenomas | Colon diverticulosis; Hemorrhoids GII. | / | / |
| HRA10 | F | 59 | Ascending colon | High Risk adenomas | Hypertension. | / | / |
| HRA11 | M | 57 | Ascending colon | High Risk adenomas | Hypertension. | / | / |
| HRA12 | F | 59 | Descending colon | High Risk adenomas | / | / | / |
| HRA13 | M | 66 | Descending colon | High Risk adenomas | Hypertension. | / | / |
| HRA14 | M | 65 | Ascending colon | High Risk adenomas | Colon diverticulosis. | / | / |
| HRA15 | M | 50 | Descending colon | High Risk adenomas | / | / | / |
| HRA16 | M | 52 | Ascending colon | High Risk adenomas | / | / | / |
| HRA17 | M | 62 | Descending colon | High Risk adenomas | Colon diverticulosis. | / | / |
| HRA18 | M | 51 | Ascending colon | High Risk adenomas | Colon diverticulosis; Hemorrhoids GI; Hypothyroidism. | / | / |
| HRA19 | M | 67 | Descending colon | High Risk adenomas | Hypertension; Dyslipidemia. | / | / |
| HRA20 | F | 68 | Descending colon | High Risk adenomas | Hemorrhoids GI; Hypertension; Dyslipidemia. | / | / |
| HRA21 | M | 66 | Ascending colon | High Risk adenomas | Diverticulosis. | / | / |
| ADK1 | M | 69 | Descending colon | Adenocarcinomas | Angiodysplasia of minimum size; Hypertension; Dyslipidemia. | / | / |
| ADK2 | M | 66 | Descending colon | Adenocarcinomas | Hemorrhoids GI; Diverticulosis. | / | / |
| ADK3 | M | 66 | Ascending colon | Adenocarcinomas | Hypertension. | / | / |
| ADK4 | F | 63 | Descending colon | Adenocarcinomas | Hypertension; Dyslipidemia. | / | / |
| ADK5 | M | 70 | Descending colon | Adenocarcinomas | / | / | / |
| ADK6 | F | 66 | Ascending colon | Adenocarcinomas | Scleroderma. | / | / |
| ADK7 | F | 69 | Ascending colon | Adenocarcinomas | Hypertension. | / | / |
| ADK8 | F | 70 | Ascending colon | Adenocarcinomas | / | / | / |
| ADK9 | F | 40 | Ascending colon | Adenocarcinomas | / | Neoadjuvant chemotherapy: Folfiri, Cetuximab. | / |
| ADK10 | M | 77 | Descending colon | Adenocarcinomas | Hypertension. | Neoadjuvant chemotherapy: mFolfox6, Bevacizumab. | / |
| ADK11 | F | 62 | Descending colon | Adenocarcinomas | Hemorrhoids GII. | / | Neoadjuvant tomotherapy |
| ADK12 | F | 66 | Descending colon | Adenocarcinomas | / | Capecitabina, Xeloda. | / |
| ADK13 | F | 68 | Ascending colon | Adenocarcinomas | Hypertension; type II Diabetes. | Neoadjuvant chemotherapy: Folfoxiri, bevacizumab, 5-fluoruracil. | / |
| ADK14 | M | 80 | Descending colon | Adenocarcinomas | Hypertension; Chronic obstructive pulmonary disease; Benign prostatic hyperplasia. | Neoadjuvant chemotherapy: De Gramont, Bevacizumab. | / |
| ADK15 | M | 70 | Ascending colon | Adenocarcinomas | / | Neoadjuvant chemotherapy: mFolfox6, Bevacizumab/ 5-fluoruracil, Bevacizumab/ Capecitabina, Bevacizumab. | / |
| ADK16 | F | 67 | Descending colon | Adenocarcinomas | / | Folfox (5-fluorouracil + oxaliplatin) | Tomotherapy |
| ADK17 | M | 70 | Descending colon | Adenocarcinomas | / | Folfox (5-fluorouracil + oxaliplatin) | Tomotherapy |
| ADK18 | M | 55 | Descending colon | Adenocarcinomas | / | Folfox (5-fluorouracil + oxaliplatin), Bevacizumab. |  |
| ADK19 | M | 77 | Descending colon | Adenocarcinomas | / | Folfox (5-fluorouracil + oxaliplatin) | Tomotherapy |
| ADK20 | M | 73 | Ascending colon | Adenocarcinomas | Hypertension; Dyslipidemia. | Capox regimen: oxaliplatin and fluoropyrimidine | / |
| ADK21 | F | 66 | Ascending colon | Adenocarcinomas | Hypothyroidism. | Capox regimen; Capecitabina. | / |

**Table S1. Clinical data of patients and healthy people.**


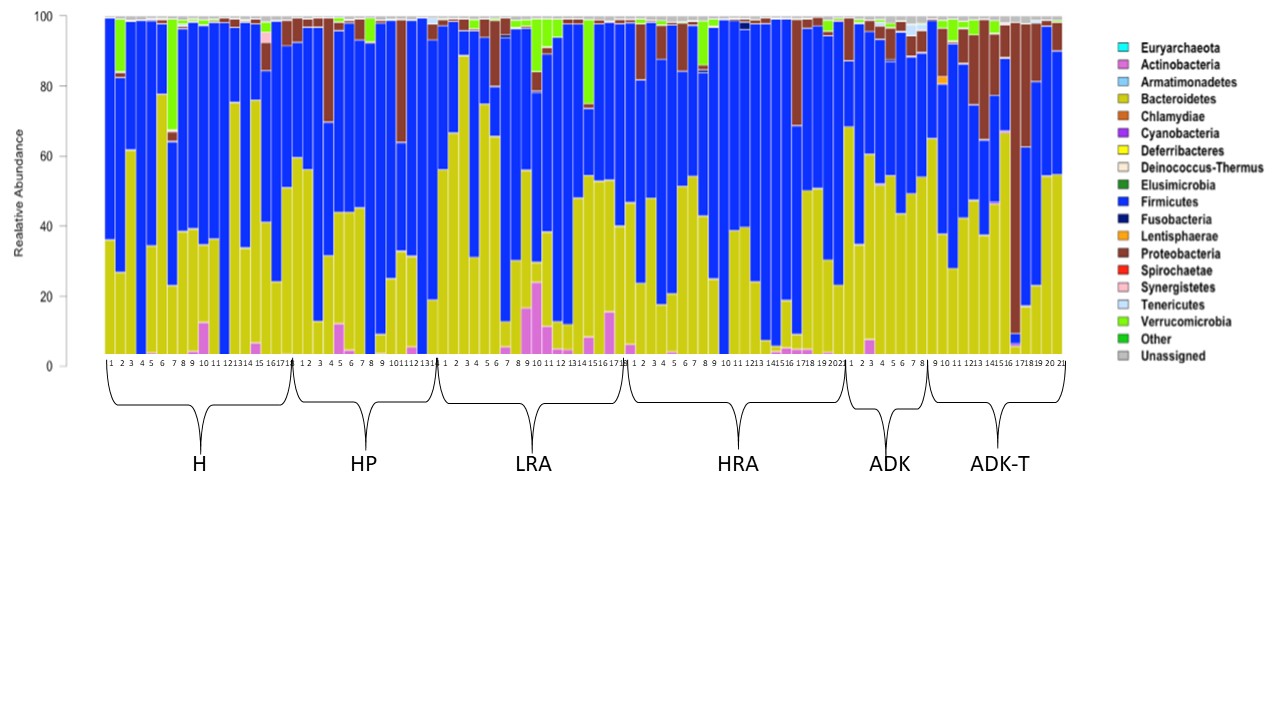


**Figure S1. Relative abundance of main bacterial phyla detected in the faecal microbiota of the enrolled subjects.**

Relative abundance of each phylum is reported. Colours are assigned for each of the detected phyla. Histograms are based on the proportion of OTUs per subject. Subjects belonging to healthy (H), hyperplastic polyps (HP), low-risk adenomas (LRA), high-risk adenomas (HRA) and adenocarcinomas (ADK) groups and patients with ADK who received chemotherapy and/or radiotherapy treatment (ADK-T) are reported.


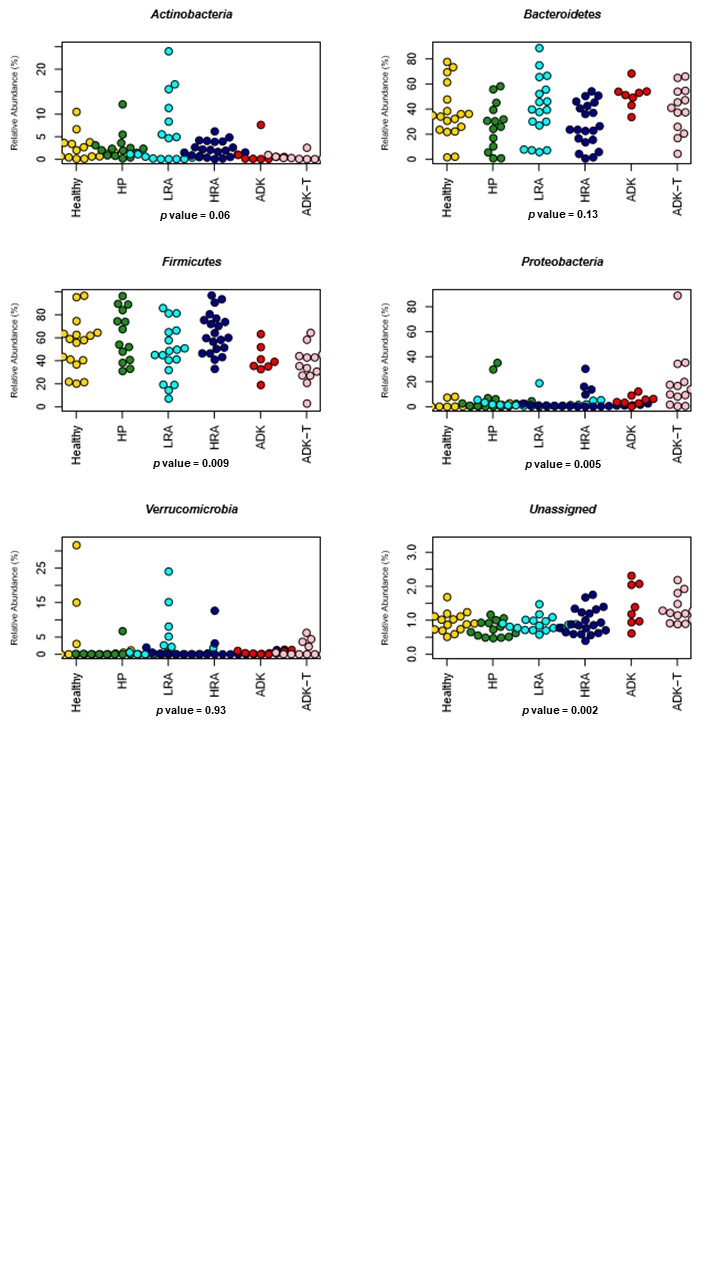


**Figure S2. Beeswarm plots of the main phyla.**

*P*-values are reported (Kruskal-Wallis test).


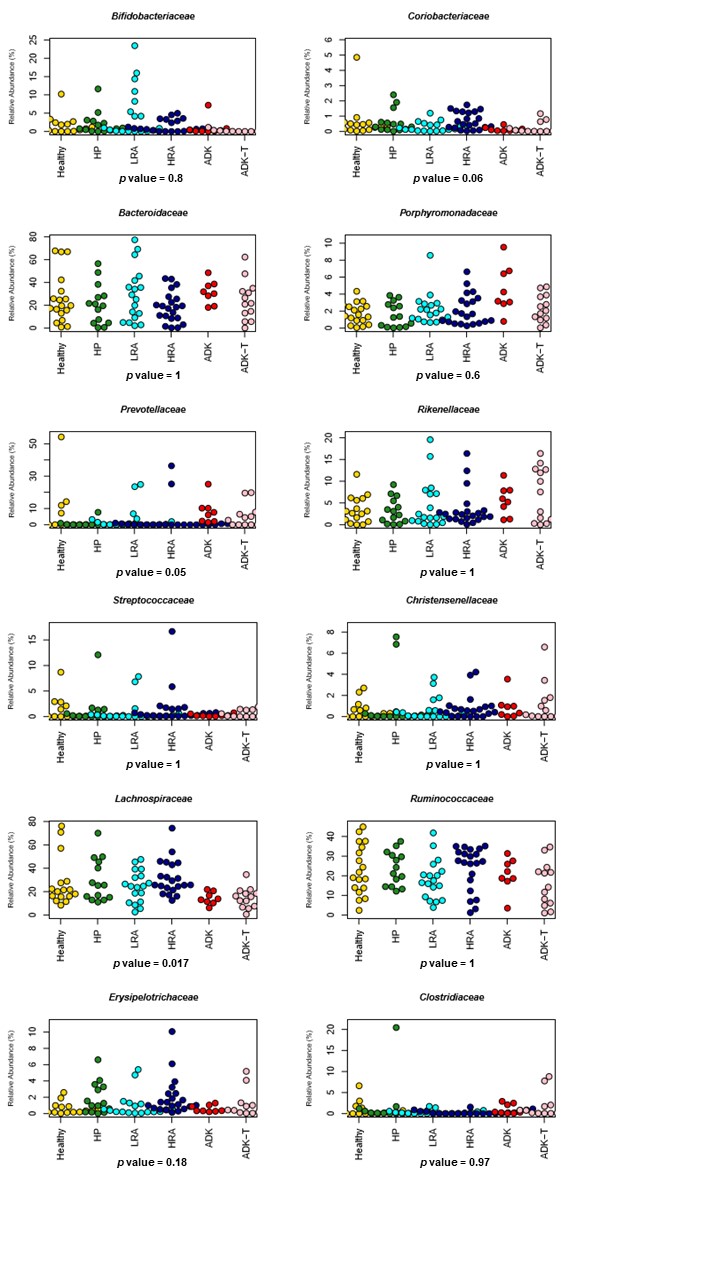


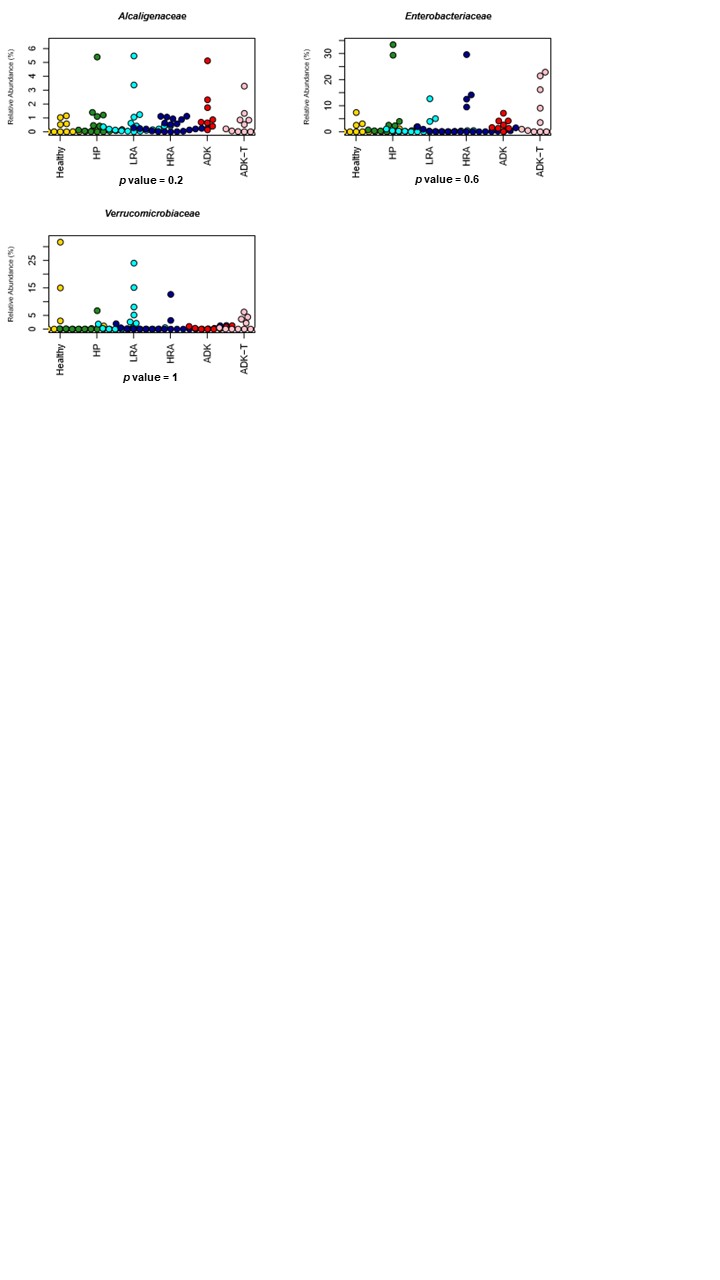


**Figure S3. Beeswarm plots of the most important detected families.**

*P*-values are reported (Kruskal-Wallis test).


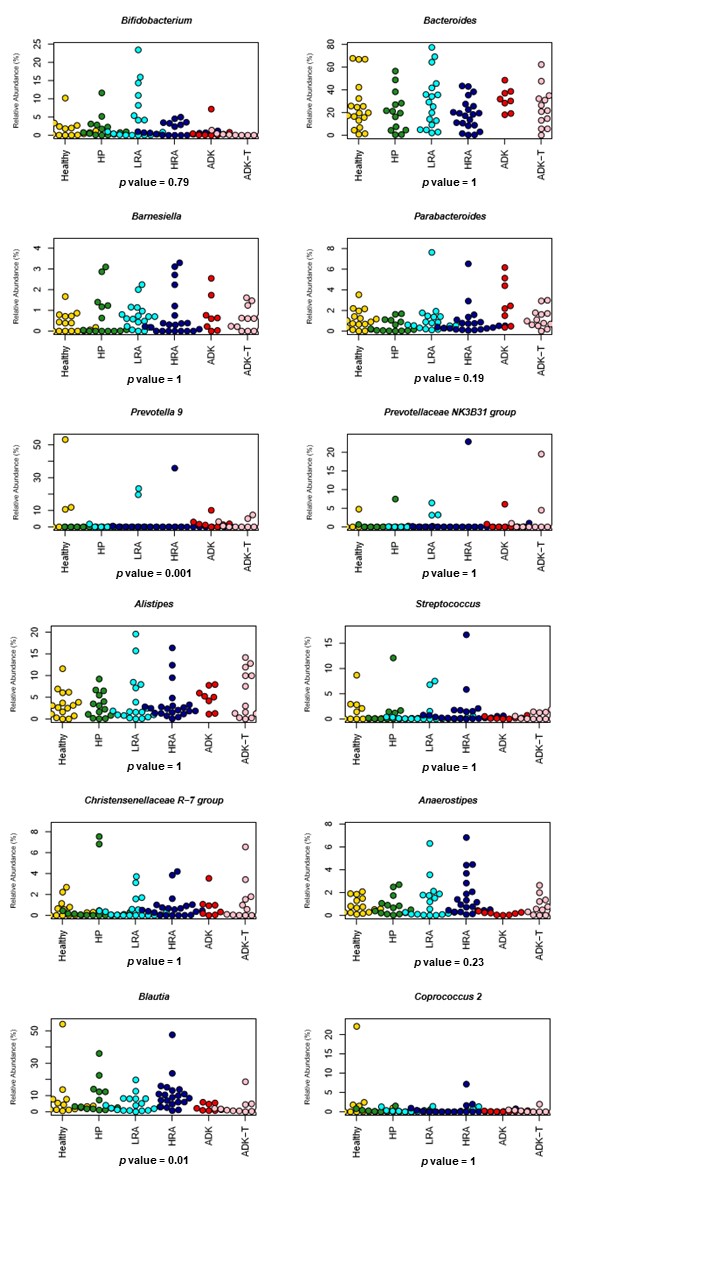


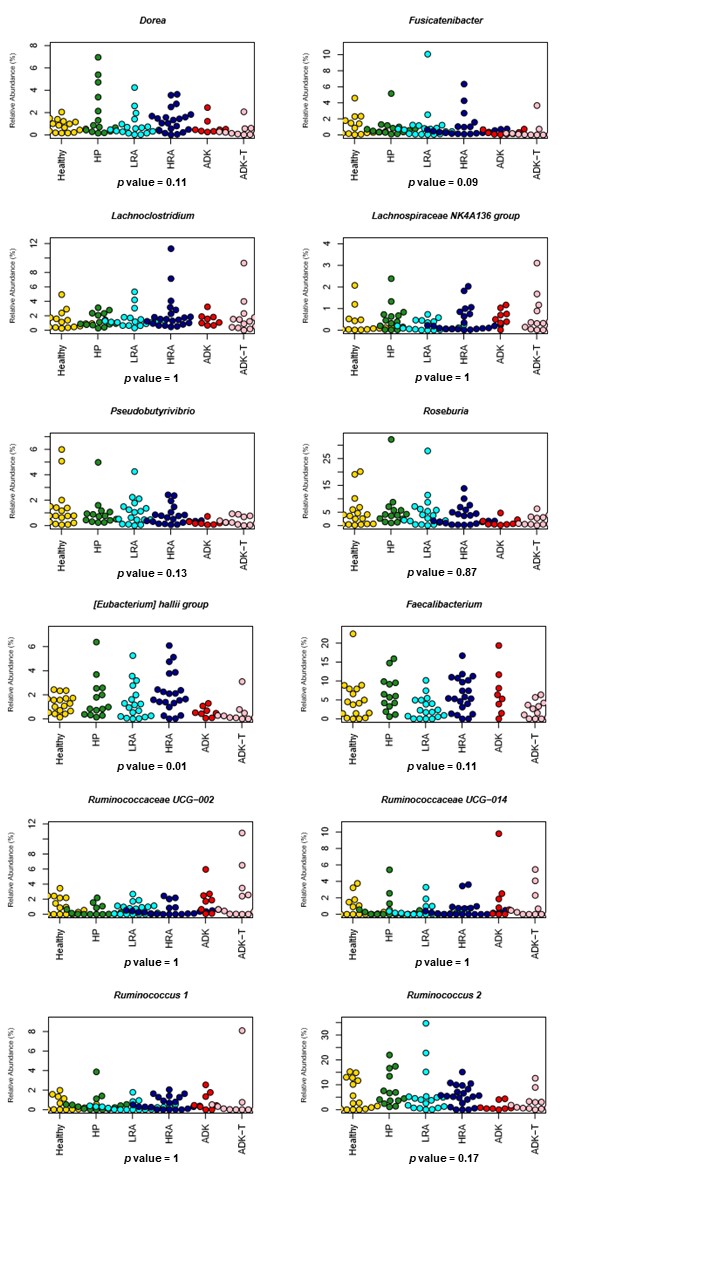


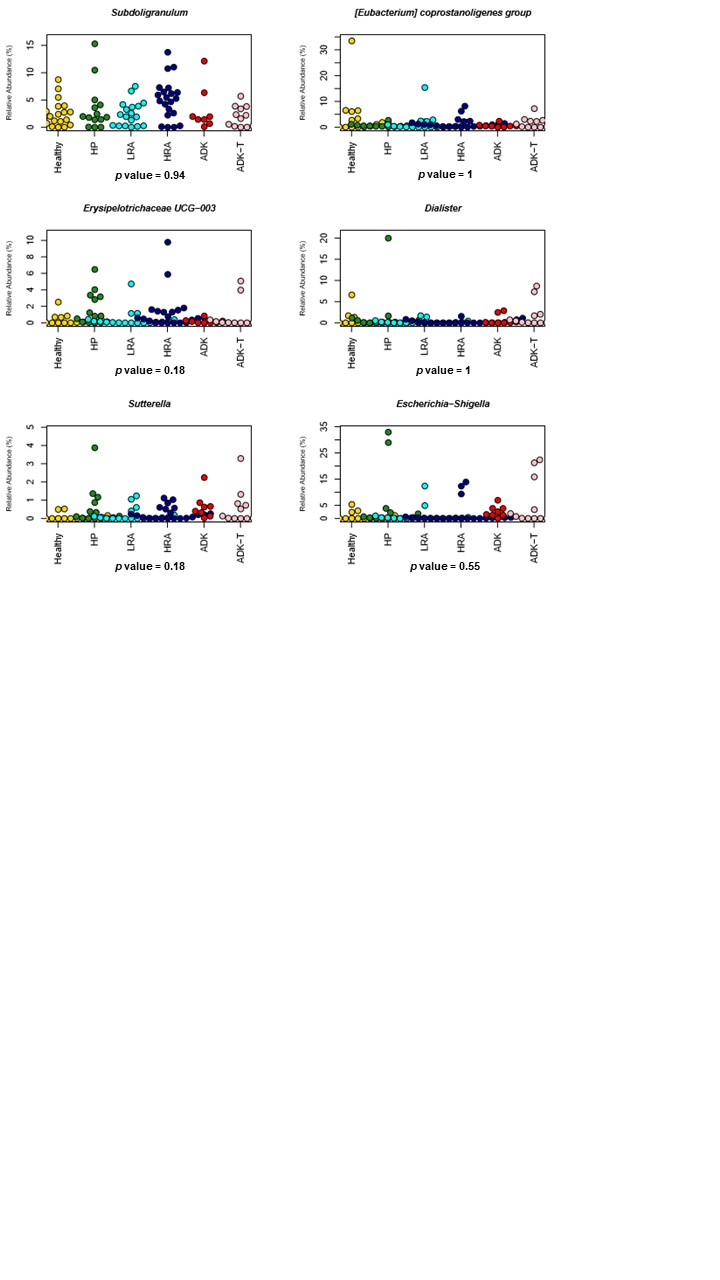


**Figure S4. Beeswarm plots of the most important detected genera.**

*P*-values are reported (Kruskal-Wallis test).

| **Sample Type (% cases)** | **Samples** | **% *Prevotella*** | **Age at diagnosis** | **Lesions location** | **Comorbidities** | **Drugs/ Chemotherapy/ Radiotherapy** |
| --- | --- | --- | --- | --- | --- | --- |
| H  (16.7%) | H3 | 53.2 | 67 | - | Hemorrhoids. | - |
|  | H11 | 11.9 | 59 | - | Hemorrhoids; colon diverticulosis. | - |
|  | H14 | 10.7 | 68 | - | Hypertension; dyslipidemia. |  |
| HP  (0%) | - | - | - | - | - | - |
| LRA  (15.8%) | **LRA14** | 1.8 | 53 | Descending colon | Colon diverticulosis; hemorrhoids; hypertension; dyslipidemia. |  |
|  | LRA16 | 23.4 | 67 | Ascending colon | - | - |
|  | LRA18 | 19.6 | 61 | Descending colon | - | - |
| HRA  (14.3%) | HRA7 | 35.8 | 61 | Descending colon | Colon diverticulosis; Hemorrhoids; Ischemic heart disease. | - |
|  | **HRA14** | 0.5 | 65 | Ascending colon | Colon diverticulosis. |  |
|  | **HRA17** | 0.4 | 62 | Descending colon | - | - |
| ADK  (66.7%) | **ADK1** | 3 | 69 | Descending colon | Angiodysplasia of minimum size; Hypertension; Dyslipidemia. |  |
|  | **ADK3** | 1.9 | 66 | Ascending colon | Hypertension. |  |
|  | **ADK4** | 1.3 | 63 | Descending colon | Hypertension; Dyslipidemia. |  |
|  | ADK6 | 10.7 | 66 | Ascending colon | Scleroderma. |  |
|  | **ADK7** | 1.7 | 69 | Ascending colon | Hypertension. |  |
|  | **ADK8** | 1.2 | 70 | Ascending colon | - | - |
| ADK-T  (38.5%) | **ADK10** | 3.2 | 77 | Descending colon | Hypertension. | Neoadjuvant chemotherapy: mFolfox6, Bevacizumab. |
|  | **ADK11** | 7.3 | 62 | Descending colon | Hemorrhoids GII. | Neoadjuvant tomotherapy. |
|  | **ADK15** | 5 | 70 | Ascending colon | - | Neoadjuvant chemotherapy: mFolfox6, Bevacizumab/ 5-fluoruracil, Bevacizumab/ Capecitabina, Bevacizumab. |
|  | **ADK17** | 0.6 | 70 | Descending colon | - | Folfox (5-fluorouracil + oxaliplatin)/ Tomotherapy. |
|  | **ADK19** | 2.8 | 77 | Descending colon | - | Folfox (5-fluorouracil + oxaliplatin)/ Tomotherapy. |

**Table S2. Samples with relative abundance of *Prevotella* (> 0%) in their gut microbiota.**

The following data are indicated: relative abundance of *Prevotella* (%) and clinical data.
